# Yellow Fever

**Published:** 1855-01

**Authors:** 


Yellow Fever.—Give quinine in the early stage of depression, promptly, boldly, and in large doses. The first stage is one of diminished vital energy which quinine raises. Give 10 to 20 grains every second or third hour. Four or six hours should never elapse between one dose and another during the stage of depression; and, when necessary, give it up gradually.—*Ibid.*

